# Multi-informant reports of preschool mental health: Validation of parent and educator reports and normative data for the preschool Pediatric Symptom Checklist and PSC-17

**DOI:** 10.1186/s13034-025-00985-3

**Published:** 2025-12-09

**Authors:** Rebecca K. McLean, Lucy A. Tully, Mark R. Dadds

**Affiliations:** School of Psychology, University of Sydney, Camperdown, NSW 2050 Australia

**Keywords:** Child mental health, Screening, Assessment, Multi-informant, Educator

## Abstract

**Background:**

The prevalence of mental health problems and unmet need in preschool-age children highlight the challenge of identifying emerging difficulties using validated measures. Given the lack of existing brief screening measures for preschool-age children, especially multi-informant measures, this study examined two versions of the Pediatric Symptom Checklist (PSC) as reported by parents and educators, the *Preschool Pediatric Symptom Checklist* (PPSC) and the *PSC-17*. In line with Standards for Reporting Diagnostic accuracy studies (STARD) guidelines, this study examined the psychometric properties, scoring thresholds, and acceptability of parent-reported PPSC. It also examined the psychometric properties of educator ratings for both PSC measures and the incremental validity of educator and parent ratings.

**Methods:**

Two studies present validation evidence for two mental health measures for use with preschool-age children. Participants were a nationally representative sample of Australian parents (*n* = 1,045; study 1) and a paired sample of parents and educators (*n* = 94 dyads; study 2) of children aged 3–5 years.

**Results:**

Results supported the internal consistency, test-retest reliability, concurrent validity of the PPSC. Parents and educators indicated high levels of acceptability of both PSC measures. Results indicated parent-reported PPSC and PSC-17 significantly improved the prediction of clinician-rated functioning scores over and above educator report suggesting incremental validity for multi-informant report. Normative data for the parent-reported PPSC are presented for the first time.

**Conclusions:**

This research expands the evidence base for the validity, reliability and acceptability of the parent and educator-report PPSC and PSC-17 measures as utilised with young children. Although further research is required, this research contributes new evidence, including incremental validity and normative data, to increase the clinical utility of both PSC measures.

**Supplementary Information:**

The online version contains supplementary material available at 10.1186/s13034-025-00985-3.

Despite the early onset and prevalence of mental health (MH) problems in preschool children aged under 5 years [1], young children are less likely to access services for their MH concerns compared to adolescents [2]. Prevalence estimates of child MH problems in preschool-aged children vary widely from 7.1 to 26.4% [3–5] and without access to services or treatment, problems can remain stable, persist into adulthood and have long-term negative ramifications [6–8]. Early identification of MH problems can improve outcomes [9]; however, in order to identify children in need of intervention, validated measures suitable for use in the preschool population, using multi-informant reports from primary caregivers, are needed. The *Preschool Pediatric Symptom Checklist* (PPSC; [10]) and *Pediatric Symptom Checklist-17* (PSC-17; [11]) are broad-based child MH measures, but there are significant gaps in the research supporting these measures with parents and educators of preschool-aged children; they require further validation and normative data in order to have greater clinical utility, and be adopted in research and clinical practice. This study presents new validation evidence for the PPSC and PSC-17 for parent and educator reports for preschool-age children and provides normative data for the PPSC for the first time.

## Universal screening

The prevalence of problems in preschool-age children highlights a need to identify MH difficulties early so that children can receive intervention. One tool for early identification is a population-based approach called universal screening, which is also known as systematic monitoring, routine surveillance, or wellbeing check-ins. Screening involves the use of standardised measures, such as the Patient Health Questionnaire [12] and Spence Children’s Anxiety Scale [13] to identify children who may be at risk of MH problems. The measures are rated by single or multiple informants, and generally quick and inexpensive to implement [14]. Screening is particularly important in the early years when MH problems are less entrenched and severe [15].

Screening measures or check-ins can be useful for parents, health professionals, and educators. Completion of wellbeing check-ins may lead to an opportunity to discuss child MH, since research suggests few parents or educators will raise concerns with each other spontaneously, and instead will wait for other caregivers to alert them to potential concerns [16, 17]. This emphasises the importance of developing multi-informant tools that are considered acceptable by parents and educators.

## Multi-informant screening utilising parents and educators

Whilst reports between raters are often not highly consistent, multi-informant assessment is a hallmark of developmental psychology and essential for early identification [18, 19]. Multi-informant measures are needed to identify children at risk of MH problems; however, two recent systematic reviews have highlighted there are few measures validated for use with preschool-aged children either with single or multiple raters [20, 21]. In order to allow multi-informant reports, there is a need, therefore, to establish psychometrically sound measures for young children that can be used with parents and educators.

Early childhood education and care (ECEC) educators’ perspectives of child MH are valuable, as they observe children in naturalistic play for many hours and can compare child MH with a referent group of peers [22, 23]. Whilst medical professionals are increasingly using screening measures in their practice [24], educators often lack MH training and knowledge around measure use [25]. Yet, educator reports are one of the primary pathways of identification of MH problems in young children [26] and educators are a primary source of advice when parents are help-seeking for their child [27]. There is, therefore, a need to provide parents and educators with measures that will help them identify children at risk of MH difficulty.

Whilst adding educator perspectives to parent-report measures has the potential to increase the validity of screening, it can also raise the possibility of discrepant informant ratings [18]. Although discrepancies between informants may appear disadvantageous, they can in fact offer important, domain-specific information about a child’s behaviour informed by the informant’s context [28]. When multiple perspectives about a child are captured from various contexts like the home and preschool, the ideal model of child assessment is more likely to be obtained [29]. Moreover, incremental validity is important for multi-informant measures as it provides an indication of how much each informant’s perspective contributes to a specified outcome and whether additional perspectives are justified. However, incremental validity of multi-informant measures for preschool children has not been well researched. A recent systematic review identified only one study investigating incremental validity of two measures; a significant improvement in identification of child MH problems was found when educator ratings were added to parent ratings for the PSC-17 and Behavior Assessment System for Children-3 Behavioral and Emotional Screening System [21]. The lack of research in this area reveals a need to understand the incremental value of collecting data from multiple caregivers.

## The Pediatric Symptom Checklists

Whilst there are several MH screening measures, which have been identified in previous reviews of the literature, only a few have been validated for use with preschool-age children and these measures have varying psychometric strength [20, 30]. Psychometric properties such as internal consistency, test-retest reliability, inter-rater reliability, and predictive validity are important for ensuring measures are free from measurement error and are suitable for use with the population in which they were tested. Two *Pediatric Symptom Checklists* (PSCs) have been recommended for use with preschool populations: the PSC-17 [11] and PPSC [10]. Both are brief, free in digital and paper-based formats, and easy to score. They produce total scores and subscales based on internalising, externalising and attention problems. The PSC-17, which is widely used, was developed for older children, but has recently been validated for use with children aged 3–5 years [31]. However, investigations of educator report for the PSC-17 are limited [32–34]. Previous studies have investigated the factor structure of educator-rated PSC-17, inter-rater reliability and incremental validity in the preschool environment [32, 34, 35], however, other facets of reliability and validity of educator report with a paired sample of parents and educators have not been investigated for the PSC-17. Further, the convergence of educator-report with diagnostic clinical interviews has not been investigated for this measure. The 18-item PPSC is a related measure, adapted from the PSC-17 and specially developed for preschool-aged children 18 months–5.5 years, with developmentally appropriate item wording and a fourth subscale called “Parenting Challenges”, which assesses parent difficulties across six items. However, the developers of this scale caution against interpreting any of the subscales, due to concerns over reliability, and instead suggest use of the total score only. Whilst previous research on the PPSC features diverse samples [36, 37], there is no normative data and the measure has not yet been examined with educators, limiting its clinical utility. The PPSC has strong internal consistency, Cronbach’s alpha 0.80–0.92, and good test-retest reliability (intraclass correlation coefficients [ICC] = 0.75) after four weeks [10, 38]. Classification accuracy or predictive validity is important for screening measures; the PPSC has high sensitivity (0.87 − 1.00) when tested against the Child Behaviour Checklist (CBCL) and the Ages & Stages Questionnaires [10]. Recent research reported moderate predictive validity of sensitivity 0.75, specificity 0.77, Positive Predictive Value (PPV) 0.60, Negative Predictive Value (NPV) 0.99 for the PPSC compared to International Classification of Disease scores after 1 year [39].

Clinical thresholds or cut-off points in screening measures determine the classification of children and their level of risk for MH problems [40]. Determining appropriate clinical thresholds is key to early identification. If thresholds are too low, healthcare services risk being overwhelmed and parents unnecessarily concerned; too high and at-risk children may be overlooked for intervention [40]. It is, therefore, important for researchers to determine appropriate thresholds and normative data based on the prevalence of child psychopathology and the population of interest. No normative data have been established for the PPSC. Whilst the PSC-17 has been the focus of a number of studies [31], more limited research on the psychometric properties of the PPSC warrants further attention. Thus, this study includes an examination of both PSC measures that are appropriate for use with preschool populations.

## Acceptability

Acceptability in terms of whether a measure is perceived as easy to comprehend, helpful or useful can affect implementation of screening [15, 41]. Acceptability may increase uptake and implementation of screening programs and therefore, it is an important aspect to investigate if measures are to be adopted as part of assessment and screening practices [42]. The acceptability of the PPSC, as reported by any user, has not been explored. Investigating whether educators find the PSC measures acceptable is important if these measures are to be adopted in ECEC services.

## The current study

No research has investigated the PPSC with educators, little research has investigated incremental validity for either PSC measure and no studies have investigated acceptability of the PPSC. Thus, the overall aims were to examine validity and establish normative data for parent-reported PPSC, examine the validity of educator-reported PPSC and PSC-17, and examine acceptability of parent- and educator-reported PPSC.

Two studies were conducted with two cohorts. The first study examined the validity and acceptability of parent-reported PPSC, and the second study examined the validity of educator-reported PPSC and PSC-17, and incremental validity. Analyses were conducted using SPSS 29.0.1.0 [43].

### Study 1

Study 1 aimed to: (1) examine the psychometric properties of parent-reported PPSC, in terms of internal consistency, test-retest reliability, concurrent and predictive validity, and to also examine scoring thresholds; (2) establish normative data for PPSC in Australia; and (3) examine the acceptability of this measure.

### Method

#### Procedures

**Fig. 1:**
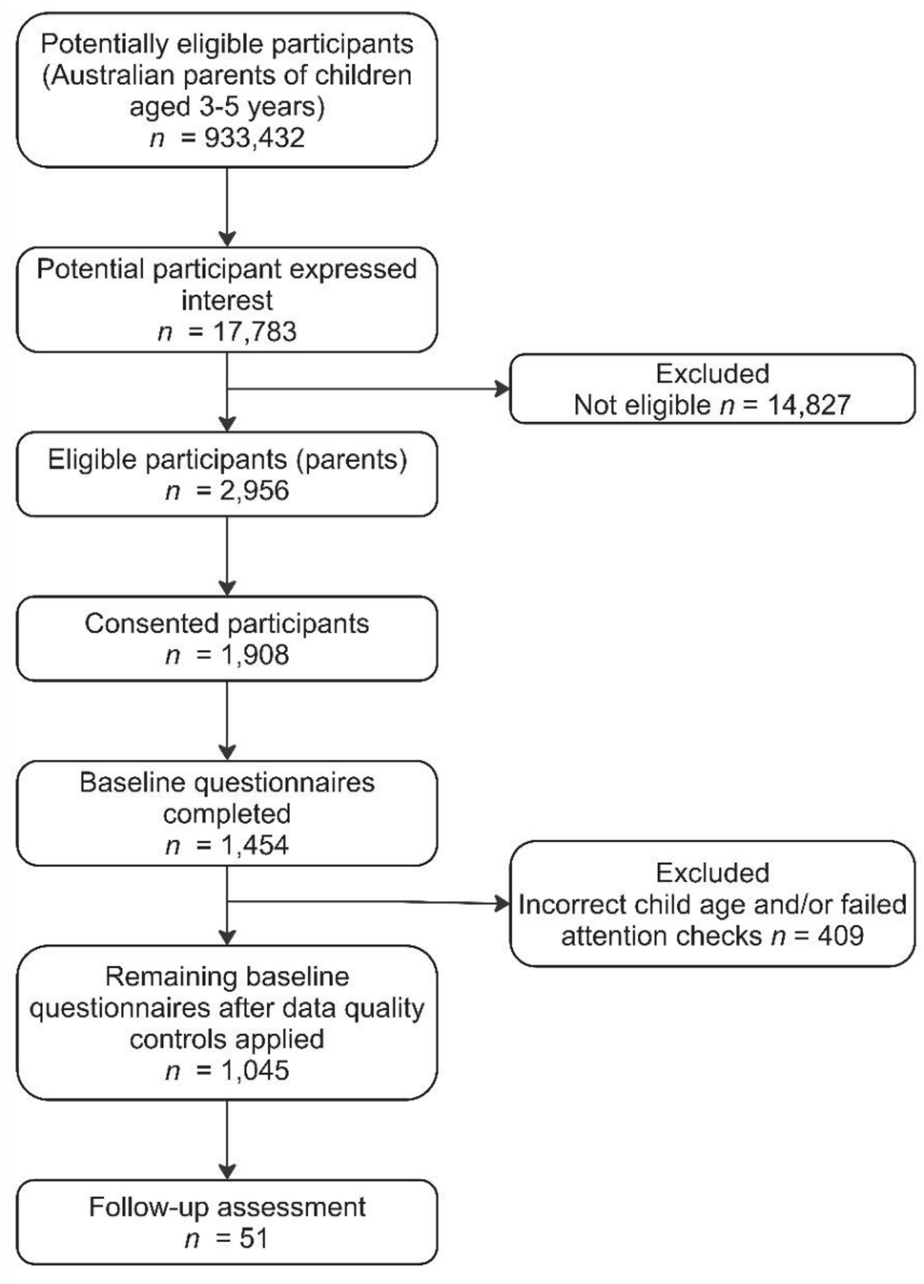
Flow of participants through Study 1

Whilst there are no set conventions for sample sizes for normative data, this study aimed to recruit a large, representative sample from the Australian population using an online research panel. Participants were recruited through an Australian, online research panel and sampled to represent families based on key socio-demographic characteristics from national census data (i.e., household income, marital status, and residential location) using segmented quotas. After sampling, parent demographic details including household income, marital status, and residential location (metropolitan, regional/rural) were compared to census data to ensure key characteristics of the sample aligned with the broader population [44–46]. Adult parents or caregivers of a child aged 3–5 years, who lived in Australia and understood English completed baseline measures (*N* = 1,045), which were counterbalanced in order of completion. Participants who completed baseline questionnaires were invited to participate in a second assessment. To evaluate test-retest reliability, concurrent and predictive validity, a sub-sample of participants (*n* = 51) completed a second set of questionnaires on average 35 days after baseline. Participant flow is presented in Fig. 1. See supplementary material for additional details about recruitment procedures.

#### Participants

Mean child age was 3.87 years (*SD* = 0.78) and 46.89% were female. Child ethnicity was predominantly Caucasian, but diverse (See supplementary material for additional details about recruitment procedures). Parents were aged 36.30 years on average (*SD* = 8.10); 66.6% of parents identified as female; and 84.1% of parents were married or in *de facto* relationships.

#### Measures

In addition to the PPSC, parents’ views on the acceptability of the PPSC were assessed using four items adapted from a parent-report measure developed by Hawes et al [47]. Responses were collected on a 5-point Likert response scale from *not at all/strongly disagree (1)* to *very/strongly agree (5)* . Items index confidence in ratings (“Based on your knowledge of your child, how confident are you in the responses/ratings you have given today as part of the Checklist?”), the appropriateness (“How appropriate did you find the wording of the questions, i.e., were the questions appropriate for your child’s age or developmental stage?” and “The Checklist asks important questions about my child’s mental health”) and clarity of item wording (“How clear did you find the wording of the questions, i.e., were they easy to understand?”).

The CBCL for 1.5-5 years [48] was used as a criterion measure at the second assessment point to establish concurrent and predictive validity with PPSC. The CBCL is a comprehensive, 99-item measure of child MH. Cases were classified as borderline, clinical, or at-risk including both borderline and clinical cases. The CBCL has strong psychometric properties and is the most common criterion measure in classification accuracy studies [30].

#### Analytic plan

To examine the first aim, Cronbach’s alpha for internal consistency and ICC for test-retest reliability were assessed. ICC estimates and their 95% confident intervals were calculated based on a single rating, absolute-agreement, 2-way mixed-effects model. To assess concurrent validity with the CBCL, Pearson correlations were calculated. PPSC scores were classified into at-risk categories using the recommended scoring threshold. Receiver operator characteristic (ROC) analysis was used to evaluate concurrent validity based on categorical risk classifications and to examine recommended thresholds. Low cell counts meant assumptions were violated for Chi-Square tests. Fisher’s Exact Test was not significant thus we were unable to analyse the predictive validity of the PPSC compared to the CBCL clinical or at-risk cases as intended. Psychometric properties were evaluated according to the rubric developed by Youngstrom et al [49] and De Los Reyes and Langer [50].

To address the second aim, normative data was determined by reporting scoring cut-offs for the 90th (borderline) and 95th percentile (at-risk) for overall age and gender (*N* = 1,045). To address the third aim of acceptability, frequency distributions were examined (*n* = 51).

Since the online survey required input for all items, there was no missing data.

### Results

#### Reliability and validity

Excellent internal consistency (α = 0.90) and adequate test-retest reliability was found for PPSC total scores (ICC = 0.78, CI 0.65–0.87).

PPSC total scores strongly and positively correlated with CBCL total scores at the bivariate level demonstrating concurrent validity, *r* = 0.86, *p* < 0.001, *n* = 51.

ROC analysis examined the association between PPSC and CBCL at-risk case classifications. The area under curve (AUC) for PPSC total scores was 0.89 with a standard error of 0.09. This was significant at *p* < 0.001 (CI 0.72–1.06).

ROC analysis identified an alternative clinical threshold of 19.5 for the PPSC, which maximised sensitivity and specificity. For ease of scoring, the alternate score was rounded down and analysed at 19, which identified 10.0% (*n* = 104) of the sample as at-risk. Chi-square tests of independence indicated an adequate number of cell counts using the alternate threshold. An alternate clinical threshold using a criterion of CBCL clinical cases produced sensitivity of 83.3, specificity 95.6, PPV 71.4, NPV 97.7, and AUC 0.89 (standard error 0.09), *p* < 0.001.

#### Normative data

The mean, standard deviation and ranges for total scores and subscales were calculated. Using the recommended scoring threshold, 47.4% of the sample (*n* = 495) were identified as at-risk. Table 1 presents scores for the highest-ranking cases by age and gender.

**Table 1:** Mean scores and banding for PPSC subscales and total scores

		3–5 years		
		(*N* = 1,045)	Male (*n =* 554)	Female (*n =* 490)
Internalising	M	3.06	3.28	2.81
	SD	2.47	2.56	2.35
	Range	0–12	0–12	0–12
	Possible range	0–12		
	α	0.777	0.790	0.757
	At-risk (top 5%)	8	8	7
	Borderline (top 10%)	7	7	6
Externalising	M	1.30	1.56	1.01
	SD	1.65	1.82	1.39
	Range	0–8		
	Possible range	0–8	0–8	0–8
	α	0.776	0.805	0.705
	At-risk (top 5%)	4	5	4
	Borderline (top 10%)	4	4	3
Attention	M	1.95	2.21	1.67
	SD	1.72	1.81	1.57
	Range	0–6	0–6	0–6
	Possible range	0–6		
	α	0.808	0.825	0.771
	At-risk (top 5%)	6	6	5
	Borderline (top 10%)	5	5	4
Parenting challenges	M	3.24	3.55	2.88
	SD	2.91	3.02	2.75
	Range	0–12	0–12	0–12
	Possible range	0–12		
	α	0.834	0.844	0.816
	At-risk (top 5%)	9	10	9
	Borderline (top 10%)	8	8	7
Total scores	M	9.22	10.21	8.10
	SD	6.81	7.28	6.07
	Range	0–36	0–36	0–36
	Possible range	0–36		
	α	0.900	0.911	0.877
	At-risk (top 5%)	23	25	20
	Borderline (top 10%)	18.4	20	16.9
	PPSC case rate (% positive)	47.4	53.1	40.8

Data not reported for one child identified as non-binary gender

#### Acceptability

In response to questions of acceptability, most parents agreed or strongly agreed the PPSC asked important questions about their child’s MH (86.3%), found the PPSC to be very or quite appropriate (86.3%, *n* = 44) and very or quite clear (96.1%). Most parents were very or quite confident in the ratings they provided (96.1%).

### Study 2

Having produced new validation evidence for parent-reported PPSC in study 1, study 2 sought to extend measure utility by validating educator-reported PPSC and PSC-17 and to examine the incremental value of multi-informant ratings by recruiting parents and educators.

The aims of study 2 were to: (1) examine the psychometric properties of educator ratings of the PPSC and PSC-17 in terms of internal consistency, test-retest reliability, inter-rater reliability, predictive validity, concurrent validity and convergence with diagnostic clinical interviews; (2) examine the incremental validity of parent- and educator-report PPSC and PSC-17 scores using criterion measures including the parent-report CBCL and clinician-rated Children’s Global Assessment Scale (CGAS); and (3) examine acceptability of the measures amongst educators.

### Method

#### Procedures

An *a priori* statistical power calculation to estimate an appropriate sample for analysing measurement stability and validity was performed using G*Power 3.1.9.7 [51]. With alpha of 0.05, power 0.80, and an effect size of 0.12, the calculated sample size was 93. Participants were recruited via ECECs and social media advertisements. Adult parents or caregivers of a child aged 3–5 years, who lived in Australia, and understood English were asked for permission to contact their child’s educator, as part of the consent process. Eligible educators were adult educators of children aged 3–5 years, who worked in ECEC, had internet access, understood English and knew the child well enough to complete questionnaires about their MH. The final paired sample of parents and educators who completed baseline questionnaires was 94.

**Fig. 2:**
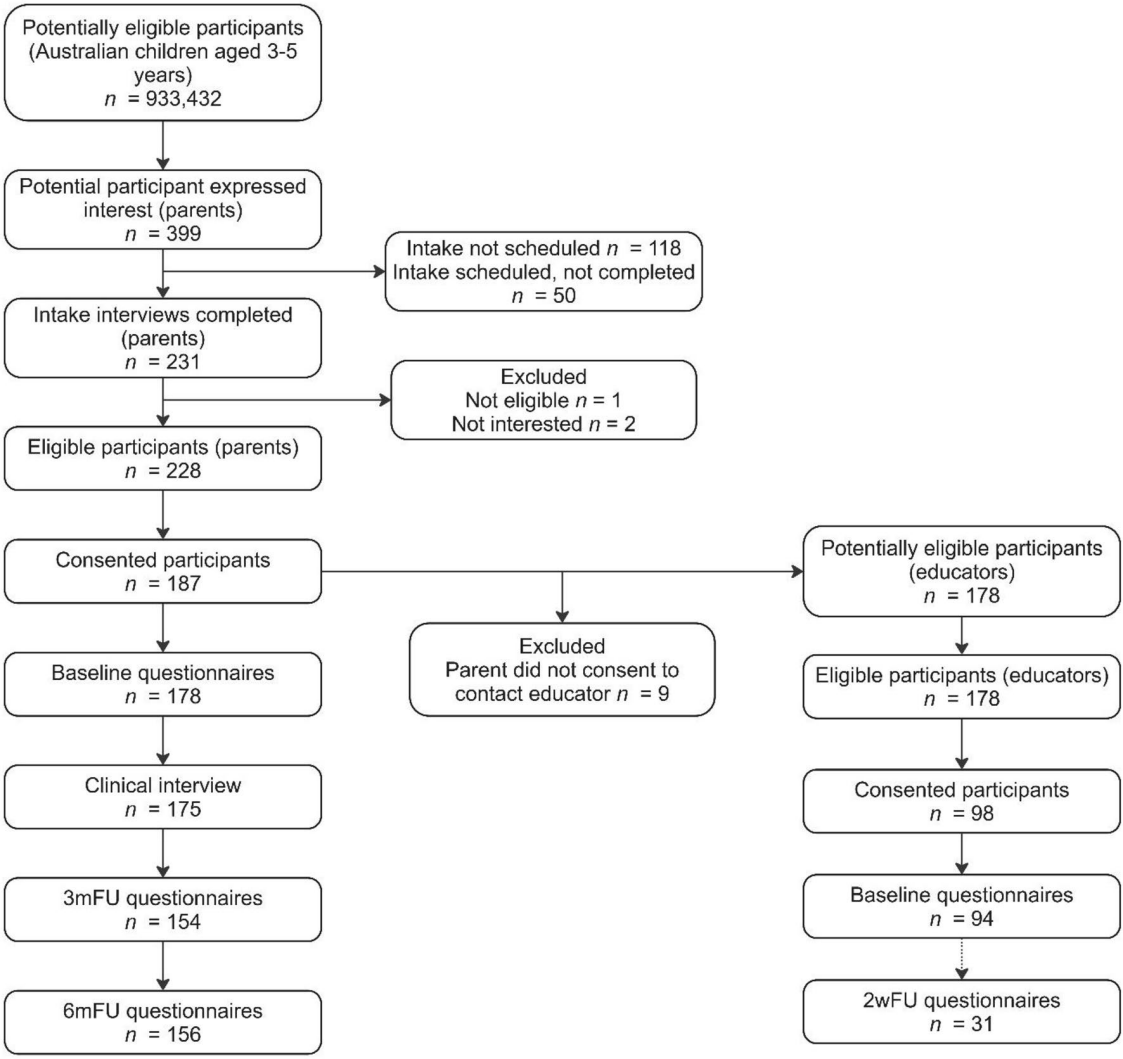
Flow of participants through Study 2

Participants completed measures which were counterbalanced in order of completion at each timepoint. Parents completed baseline, 3-month (3mFU) and 6-month follow-up (6mFU) questionnaires. Within two weeks of baseline, parents completed one clinical interview (2wFU), and educators completed baseline questionnaires. Within two weeks of educator baseline, a sub-sample of educators (*n* = 31) completed a second set of questionnaires to test test-retest reliability. Educators who completed baseline questionnaires were invited to participate in the second assessment until the target recruitment for the sub-sample was reached. Participant flow is presented in Fig. 2.

#### Participants

Children were aged 3.65 years on average (*SD* = 0.67) and 38.3% female. Child ethnicity was predominantly Caucasian (74.5%), followed by 16% other ethnicities. Educators were predominantly female (97.9%) with a mean of 13.13 years ECEC work experience (*SD* = 8.89). Most educators indicated that they were *very* or *quite* familiar with the child (89.4%, *n* = 84) and had known the child between 2 and 54 months (*M* = 13 months). See supplementary material for additional participant details.

#### Measures

Educators’ views on the acceptability of measures were assessed at baseline using the same measure as reported in Study 1.

Parents completed the CBCL at baseline, 3mFU and 6mFU. The CBCL at baseline and 3mFU was used in this study as a criterion measure to investigate convergent and predictive validity, and as an outcome measure for incremental validity.

Clinicians, who were blind to parents and educators’ questionnaires responses, conducted clinical interviews with parents using the *Diagnostic Interview Schedule for Children*,* Adolescents and Parents* (DISCAP-V; [52]) to assess child psychiatric disorder and the *Children’s Global Assessment Scale* (CGAS; [53]) to assess global functioning. The DISCAP-V is a semi-structured interview guide corresponding to common childhood behavioural and affective diagnostic categories. Clinicians rated the severity of the diagnostic presentation (0–6). Those with a severity rating 4 and above were considered to meet criteria for the specified disorder. The CGAS is an outcome measure of a child’s general functioning rated on a continuum (0–100); higher scores indicate higher levels of health and superior functioning, while lower scores indicate illness and gross impairment. Parents were blind to clinician ratings.

Clinicians were clinical psychology interns at a university research clinic, registered general and clinical psychologists, all under the supervision of senior clinical psychologists. Inter-rater reliability for a subset of clinical interviews (*n* = 24) was assessed using a secondary team of clinicians, who were blind to the primary clinician’s ratings. Diagnostic ratings correlated highly (*r* = 0.79, *p* < 0.001). CGAS scores moderately correlated (*r* = 0.57, *p* = 0.004). There were no significant differences in ratings.

Educators’ familiarity with the child was assessed using five items such as how frequently they care for the child, how long they have known the child, level of familiarity and strength of relationship with the child. Responses were collected on a 5-point Likert response scale from *not at all/strongly disagree (1)* [] to *very/strongly agree (5)* [].

Parent-report PPSC was completed at baseline, 3mFU and 6mFU. One item differed for educator-report PPSC: “Is it hard to take the child out in public?” with an additional response option, *Not applicable*. Scoring for this response option was coded as 0. Items were summed to produce a total score. Educators completed this measure at baseline and 2wFU.

The PSC-17 was the same for parents and educators. Parents completed this measure at baseline, 3mFU and 6mFU and educators at baseline and 2wFU.

#### Analytic plan

The following sub-samples of participants were used to analyse the data: baseline educator (*n* = 94), educator 2wFU (*n* = 31), baseline parent (*n* = 178), parent 2wFU (*n* = 175, and parent 3mFU (*n* = 154). To examine the first aim, Cronbach’s alpha was calculated to assess reliability using baseline educator assessment, ICCs were assessed for test-retest (baseline educator and 2wFU) and inter-rater reliability, and Pearson’s correlations for convergent validity with CBCL total scores (baseline parent and educator). ICC estimates and 95% confident intervals were calculated based on a single rating, absolute-agreement, 2-way mixed-effects model. To assess inter-rater agreement, Cohen’s κ was calculated using case classifications (baseline parent and educator). The two PSC measures were classified using recommended thresholds. To test concurrent validity with the DISCAP, a series of binary logistic regressions were conducted (baseline educator and parent 2wFU). To assess predictive validity, first cell counts for Chi-Square tests and Fisher’s Exact Test were checked to be valid. Baseline educator PPSC and PSC-17 ratings were then compared to parent-rated CBCL clinical cases at 3mFU. Psychometric properties were evaluated according to the same rubric as utilised in Study 1.

To address the second aim, a series of hierarchical multiple regressions were conducted to assess incremental validity (baseline educator, baseline parent and parent 2wFU). To address the third aim of acceptability, frequency distributions were examined for educators at baseline.

Since the online survey required input for all items, there was no missing data for baseline measures for parents and educators, and educator 2wFU. Listwise deletion was used for two cases where parents did not attend clinical interviews. Pairwise deletion was used for parent-reported data missing at 3mFU (*n* = 13).

### Results

#### Reliability and validity

Internal consistency was excellent for baseline educator-reported PPSC (α = 0.91) and good for PSC-17 total scores (α = 0.88). Test-retest reliability for educator-reported PPSC was excellent (ICC = 0.91; CI 0.82–0.96, *p* < 0.001) and good for the PSC-17 (ICC = 0.80; CI 0.66–0.89, *p* < 0.001) for baseline and 2wFU assessments. Inter-rater reliability between baseline parent and baseline educator ratings was small for the PPSC (ICC = 0.33; CI 0.14–0.49, *p* < 0.001) and PSC-17 (ICC = 0.34, CI 0.15–0.50, *p* < 0.001).

Convergent validity was weak and insignificant for baseline educator-reported PPSC and baseline parent-reported CBCL total scores, *r =* 0.19, *p* = 0.068, and baseline educator-reported PSC-17 total scores, *r* = 0.19, *p* = 0.067. Cohen’s κ was run to determine if there was agreement between at-risk baseline educator-reported PPSC and baseline parent-reported CBCL at-risk cases, κ = 0.15, CI − 0.05–0.36, *p* = 0.123. When at-risk baseline educator-reported PPSC cases were compared to baseline parent-reported CBCL clinical cases, there was significant, though low, agreement, κ = 0.19, CI − 0.01–0.40, *p* = 0.040. At-risk baseline educator-reported PSC-17 cases compared to baseline parent-reported CBCL at-risk cases found κ = 0.11, CI − 0.11–0.33, *p* = 0.275, and to baseline parent-reported CBCL clinical cases κ = 0.09, CI − 0.14–0.33, *p* = 0.360.

To test concurrent validity, a series of univariate binary logistic regressions were performed with baseline educator-reported PPSC and PSC-17 total scores, and at-risk classifications as the predictors and any type of DISCAP diagnosis as the outcome (*n* = 92). None of the models were significant as presented in Table 2. Neither PSC significantly contributed towards diagnosis.

**Table 2:** Tests of convergence for educator-report PSC measures with DISCAP diagnoses

Predictor variables Reference: DISCAP diagnosis *n* = 92	χ2, significance	Nagelkerke *R*^2^	Wald
PPSC total scores	1.77, *p* = 0.183	0.03	1.84, *p* = 0.175
PPSC at-risk classifications	1.20, *p* = 0.273	0.02	1.22, *p* = 0.269.
PSC-17 total scores	1.19, *p* = 0.276	0.02	1.21, *p* = 0.271
PSC-17 at-risk classifications	1.26, *p* = 0.268	0.02	1.31, *p* = 0.253.

Coding for case classifications: 0 = not at-risk, 1 = at-risk. Coding for DISCAP diagnosis: 0 = no diagnosis, 1 = one or more diagnoses

#### Predictive validity

The predictive validity of the educator-reported measures was tested against the CBCL using Chi-Square tests. Compared to parent-reported CBCL clinical cases at 3mFU, baseline educator-reported PPSC scores produced sensitivity 24.0%, specificity 89.3%, PPV 50.0 and NPV 72.46; and PSC-17 scores produced sensitivity 36.4%, specificity 88.6%, PPV 33.33 and NPV 89.86.

Classification accuracy as indicated by AUC was moderate for the PPSC (AUC = 0.65, CI 0.51–0.79, *p* = 0.037) and PSC-17 (AUC = 0.63, CI 0.49–0.77, *p* = 0.061).

#### Incremental validity

To test incremental validity, hierarchical multiple regressions were conducted, with three blocks of variables. First, child age and gender were entered as predictors with clinician-rated CGAS as the outcome variable, neither were significant, F (2) = 1.15, *p* = 0.321, R^2^ = 0.02. In the second model, baseline parent-reported PPSC total scores were added and showed significant improvement on the first model F (3) = 14.19, *p* < 0.001, R^2^ = 0.33. In the third model, baseline educator-reported PPSC total score was added and was significant F (4) = 11.77, *p* < 0.001. It explained an additional 2.5% of variance, however this was not a significant change, indicating no increase in incremental validity when educator reports were included.

When the order of entry was reversed and baseline educator-report PPSC scores were entered in the second model and baseline parent-report scores in the third model, both models were found to significantly improve prediction of clinician-rated CGAS scores after controlling for age and gender indicating an increase in incremental validity when parent reports were included. Hierarchical regressions were also performed with parent-reported CBCL as the outcome variable. Overall, models using the PPSC explained 35.1% in variance of CGAS scores and 50% of CBCL total scores. Table 3 presents a summary of results.

**Table 3:** Summary of hierarchical regression models for educator-report PPSC and parent-report PPSC total scores as predictors

	*R* ^2^	ΔR^2^	F	df	Sig.	B	S.E.	β	Sig.
*CGAS*									
Block 1:	0.03	0.03	1.15	2	0.321				
Age						− 0.21	0.14	− 0.16	0.134
Gender						− 0.72	2.23	− 0.03	0.748
Block 2:	0.33	0.30**	14.19	3	< 0.001**				
Age						− 0.17	0.12	− 0.13	0.144
Gender						-1.77	1.87	− 0.08	0.347
Parent-reported PPSC						-1.12	0.18	− 0.55	< 0.001**
Block 3:	0.35	0.03	11.77	4	< 0.001**				
Age						− 0.20	0.11	− 0.16	0.083
Gender						-1.97	1.85	− 0.09	0.289
Parent-reported PPSC						− 0.99	0.19	− 0.49	< 0.001**
Educator-reported PPSC						− 0.26	0.14	− 0.17	0.070
*CGAS*									
Block 1:	0.03	0.03	1.15	2	0.321				
Age						− 0.21	0.14	− 0.16	0.134
Gender						− 0.72	2.23	− 0.03	0.748
Block 2:	0.15	0.12**	5.06	3	0.003*				
Age						− 0.26	0.13	− 0.20	0.045
Gender						-1.39	2.10	− 0.07	0.512
Educator-reported PPSC						− 0.54	0.15	− 0.35	< 0.001**
Block 3:	0.35	0.20**	11.77	4	< 0.001**				
Age						− 0.20	0.11	− 0.16	0.083
Gender						-1.97	1.85	− 0.09	0.289
Educator-reported PPSC						− 0.26	0.14	− 0.17	0.070
Parent-reported PPSC						− 0.99	0.19	− 0.49	< 0.001**
*CBCL*									
Block 1:	0.01	0.01	0.61	2	0.546				
Age						0.10	0.28	0.04	0.730
Gender						4.92	4.52	0.12	0.279
Block 2:	0.49	0.48**	29.15	3	< 0.001**				
Age						− 0.002	0.20	− 0.001	0.994
Gender						7.62	3.27	0.18	0.022*
Parent-reported PPSC						2.88	0.31	0.70	< 0.001**
Block 3:	0.50	0.003	21.89	4	< 0.001**				
Age						− 0.02	0.20	− 0.01	0.904
Gender						7.47	3.28	0.17	0.025*
Parent-reported PPSC						2.97	0.34	0.72	< 0.001**
Educator-reported PPSC						− 0.19	0.25	− 0.06	0.460
*CBCL*									
Block 1:	0.01	0.01	0.61	2	0.546				
Age						0.10	0.28	0.04	0.730
Gender						4.92	4.52	0.12	0.279
Block 2:	0.05	0.04*	1.73	3	0.167				
Age						0.16	0.28	0.06	0.555
Gender						5.71	4.47	0.13	0.204
Educator-reported PPSC						0.64	0.32	0.21	0.051*
Block 3:	0.50	0.44**	21.89	4	< 0.001**				
Age						− 0.02	0.20	− 0.01	0.904
Gender						7.47	3.28	0.17	0.025*
Educator-reported PPSC						− 0.19	0.25	− 0.06	0.460
Parent-reported PPSC						2.97	0.34	0.72	< 0.001**

Table presents a summary of hierarchical regression models for baseline educator-report PPSC and baseline parent-report PPSC total scores as predictor variables with CGAS and CBCL total scores as outcome variables (*n* = 92)

*Significant at *p* < 0.05, ** *p* < 0.01

Analysis was repeated with the PSC-17 and clinician-rated CGAS as the outcome variable. After controlling for age and gender, baseline parent-reported PSC-17 was a significant predictor, F (3) = 4.75, *p* = 0.004, and explained 13.9% of variance. The third model was also significant, F (4) = 4.58, *p* = 0.002 and explained an additional 3.5% variance, however this was not a significant change in incremental validity when educator reports were included.

When the order of entry was reversed, both models were found to significantly improve prediction of clinician-rated CGAS scores. After controlling for age and gender, baseline educator-report PSC-17 explained 11.3% of variance, F (3) = 3.73, *p* = 0.014. The third model was also significant F (4) = 4.58, *p* = 0.002 and explained an additional 6.1% of variance, *p* = 0.013 indicating a significant increase in incremental validity when baseline parent reports were included. Overall, models using the PSC-17 explained 17.4% in variance of CGAS scores and 41% of CBCL total scores. Table 4 presents a summary of results.

**Table 4:** Summary of hierarchical regression models for educator-report PSC-17 and parent-report PSC-17 total scores as predictors

	*R* ^2^	ΔR^2^	F	df	Sig.	B	S.E.	β	Sig.
*CGAS*									
Block 1:	0.03	0.03	1.15	2	0.321				
Age						− 0.21	0.14	− 0.16	0.134
Gender						− 0.72	2.23	− 0.03	0.748
Block 2:	0.14	0.11**	4.75	3	0.004*				
Age						− 0.15	0.13	− 0.12	0.254
Gender						-1.97	2.14	− 0.09	0.358
Parent-reported PSC-17						− 0.85	0.25	− 0.35	< 0.001**
Block 3:	0.17	0.03	4.58	4	0.002*				
Age						− 0.19	0.13	− 0.15	0.146
Gender						-2.41	2.12	− 0.12	0.258
Parent-reported PSC-17						− 0.67	0.26	− 0.27	0.013*
Educator-reported PSC-17						− 0.36	0.19	− 0.20	0.060
CGAS									
Block 1:	0.03	0.03	1.15	2	0.321				
Age						− 0.21	0.14	− 0.16	0.134
Gender						− 0.72	2.23	− 0.03	0.748
Block 2:	0.11	0.09*	3.73	3	0.014*				
Age						− 0.25	0.13	− 0.19	0.062
Gender						-1.77	2.17	− 0.08	0.416
Educator-reported PSC-17						− 0.53	0.18	− 0.30	0.004*
Block 3:	0.17	0.06*	4.58	4	0.002**				
Age						− 0.19	0.13	− 0.15	0.146
Gender						-2.41	2.12	− 0.12	0.258
Educator-reported PSC-17						− 0.36	0.19	− 0.20	0.060
Parent-reported PSC-17						− 0.67	0.26	− 0.27	0.013*
*CBCL*									
Block 1:	0.01	0.01	0.61	2	0.546				
Age						0.10	0.28	0.04	0.730
Gender						4.91	4.52	0.11	0.279
Block 2:	0.41	0.39**	20.47	3	< 0.001**				
Age						− 0.12	0.22	− 0.05	0.584
Gender						9.66	3.58	0.23	0.008*
Parent-reported PSC-17						3.20	0.42	0.64	< 0.001**
Block 3:	0.41	0.00	15.19	4	< 0.001**				
Age						− 0.13	0.22	− 0.05	0.569
Gender						9.58	3.62	0.22	0.010*
Parent-reported PSC-17						3.24	0.45	0.65	< 0.001**
Educator-reported PSC-17						− 0.06	0.32	− 0.02	0.846
*CBCL*									
Block 1:	0.01	0.01	0.61	2	0.546				
Age						0.10	0.28	0.04	0.730
Gender						4.91	4.52	0.11	0.279
Block 2:	0.06	0.05*	1.88	3	0.139				
Age						0.16	0.27	0.06	0.562
Gender						6.47	4.50	0.15	0.154
Educator-reported PSC-17						0.78	0.37	0.22	0.039*
Block 3:	0.41	0.35**	15.19	4	< 0.001**				
Age						− 0.13	0.22	− 0.05	0.569
Gender						9.58	3.62	0.22	0.010*
Educator-reported PSC-17						− 0.06	0.32	− 0.02	0.846
Parent-reported PSC-17						3.24	0.45	0.65	< 0.001**

Tables presents a summary of hierarchical regression models for baseline educator-report PSC-17 and baseline parent-report PSC-17 total scores as predictor variables with CGAS and CBCL total scores as outcome variables (*n* = 92)

*Significant at *p* < 0.05, ** *p* < 0.01

#### Acceptability

In response to questions of acceptability, educators found the PSC measures highly acceptable. Educators agreed or strongly agreed the measures asked important questions about the child’s MH (PPSC 78.7%, *n* = 74; PSC-17 87.2%, *n* = 82), found the measures to be very or quite appropriate (PPSC 84%; PSC-17 86.1%) and very or quite clear (PPSC 94.7%; PSC-17 93.6%). Educators were very or quite confident in the ratings they provided (PPSC 90.4%; PSC-17 93.6%).

Paired sample t-tests showed there was no significant difference between educator ratings of acceptability between the PPSC and PSC-17 in terms of appropriateness or clarity. In terms of whether the measures asked important questions, educators rated the PSC-17 significantly higher on average (*M =* 3.15, SD 0.69) than the PPSC (*M =* 2.98, SD 0.73), *t*(93) = -3.05, *p* = 0.003.

## General discussion

With an aim of examining the psychometric properties of parent-reported PPSC, the first study contributed new reliability and validity evidence for the measure, finding excellent internal consistency, adequate test-retest reliability and strong concurrent validity with the CBCL total scores and case classifications. Given the high proportion of children identified as at risk in this sample using the original threshold, alongside consideration of new normative data addressing the second aim, an alternative threshold was identified as 19, which is substantially higher than the original cut-off of 9. High sensitivity and specificity using alternative thresholds surpassed benchmark standards for developmental screening measures [30, 54].

This study was the first to explore acceptability of the parent-rated PPSC—the study’s third aim. Acceptability of the PPSC by parents was very high. More than 85% of parents rated the PPSC as appropriate on all indices. Our results align with previous findings with older children across health and education settings, in which parents perceived universal MH screening as appropriate and helpful [41].

The second study complemented the first by examining the psychometric properties of educator-reported PPSC and PSC-17. Very high internal consistency and test-retest reliability was found for both measures. Unsurprisingly, inter-rater reliability between parent and educator ratings was small, but significant for both measures, which corresponds with previous findings in which there is low correspondence between informants when they observe child behaviour in different settings [18]. Specifically for the PSC-17, our findings whilst small (ICC = 0.34, CI. 15-0.50, *p* < 0.001) were higher than found in a previous study on the measure, which reported a bivariate correlation of *r* = 0.26, *p* < 0.001 [34]. This was the first study to test inter-rater reliabilities for parent and educator reports on the PPSC so there are no comparisons to make in the literature. This study’s results are comparable to other studies of agreement between parent and teacher reporters on screening measures of anxiety (*r* 0.21 − 0.32, *p* < 0.01) [55], although cross-informant agreement does vary widely across measures [21]. For analyses in which the outcome variable was parent-reported CBCL or clinician-rated diagnosis (concurrent, convergent, predictive and incremental validity), educator ratings did not perform well overall, although educator classification accuracy was moderate, as indicated by AUC analysis and educator ratings for both PSC measures had high specificity. These results may reflect informant discrepancies, possible bias of the parent-report criterion measure or criterion contamination.

Addressing the second aim of incremental validity, results indicated that incremental validity of parent report in addition to educator report was demonstrated for both PSC measures when the outcome was parent-reported CBCL scores. Educator reports did not add significant variance for the parent-reported outcome. Educator-reported PSC measures significantly improved the prediction of clinician-rated functioning scores, only when educator report was entered before report. In contexts where educators are the first gate, such as an ECEC setting, parents add significantly more variance to the model, which contrasts with previous research that found educators added significantly information on top of parent ratings for the PSC-17 [34]. Interestingly, the PSC-17 did not explain as much variance in these models, suggesting the PPSC’s items assessing parenting challenges may more closely align with impairment and functioning. These findings signal the importance of gathering parent ratings on child MH in addition to educator reports. Despite challenges facing time-poor caregivers, this research highlights the value of multi-informant assessment in young children.

To address the final aim, educators rated both measures as highly acceptable. Interestingly, educators rated the PSC-17 more highly than the PPSC in terms of whether it asked important questions; a difference not reflected in parent ratings. Despite concerns that some PSC-17 items are not well-suited to this age group, results from both studies indicate the PSC-17 was perceived by parents and educators as being age-appropriate and highly acceptable. These findings align with previous research, which found parents and educators find screening programs acceptable, and parents of preschool-age children rate the PSC-17 as an acceptable screening measure [21, 41]. Findings from the present study that both PSC measures were acceptable to parents and educators support the use of these measures with caregivers of preschool-age children.

Study strengths include the large, national sample complemented by a paired parent and educator sample. This was the first to validate an educator-report version of the PPSC and utilise best-available, gold-standard criterion such as diagnostic interview. However, the large sample could not be used for all analyses and sub-samples were small. Future research may include random sampling to ensure the generalisability of results, and inviting all educators to complete follow-up questionnaires. Community recruitment meant few clinical cases were identified in the first study, which affected predictive validity analysis. Further, parent-reported CBCL may have been a source of criterion contamination; results may have biased parent-rated predictors. This study did not include either a comprehensive educator-report questionnaire nor a clinical assessment including interviews with educators, which may have biased results towards parent reports and not sufficiently accounted for MH symptoms displayed outside the home context. Future research may mitigate this by employing comprehensive educator-report measures or having clinicians interview with educators to inform diagnostic ratings. Future studies may seek to recruit larger educator samples and replicate this study’s findings.

In terms of research implications, our findings support the PPSC as a valid, reliable and acceptable measure of child MH for preschool-age children and add weight to the suitability of using the PSC-17 in the preschool-age range with educator report. As free, brief measures, the PPSC and PSC-17 are important offerings in longitudinal research, with clear benefits enabling children to be tracked longitudinally using a single measure.

The first study found that a sizable proportion of children were at risk using the existing PPSC clinical threshold of 9 [10] suggesting this threshold is too low and may overestimate children at risk of MH problems. The existing clinical threshold identified 47.4% of preschool-age children as at risk for MH problems whereas the most recent National Survey of Mental Health and Wellbeing in Australia identified 14% of children aged 4–17 years as scoring in the clinical range of the CBCL [56]. A similar survey in Western Australia which compared prevalence in older children (12–16 years) to younger children (4–11 years) reported 16% prevalence in younger children [57]. The alternative PPSC threshold of 19 identified 10.0% of the sample as at risk. Thus, this revised clinical threshold appears to be more appropriate to use in young Australian children, and should be adopted in future research and clinical practice using the PPSC. Another important implication for determining appropriate thresholds is the level of available healthcare resources that can manage the number of children identified by screening measures as requiring early intervention [58]. Without sufficient resources to manage identified at-risk children, iatrogenic effects such as family burden and over-stretched healthcare systems, may arise as a result of widespread screening programs [58].

Current universal screening and assessment practices in the preschool-age range focus predominantly on school readiness and academic competency [59, 60], however, there is a clear need for screening to be extended to child MH given the high rates of children at risk of problems. The clinical relevance and potential application of the overall findings for both PSC measures indicate caregivers may be willing to adopt screening measures in their educational and care practice. ECEC services, which are less encumbered by issues of access and stigma [61], may be an ideal setting for early identification and can make use of educators as crucial early identifiers of child MH problems. Providing educators with validated tools as part of wider early intervention programs may help identify children in need of support. However, additional training programs are also needed to help educators communicate with parents about child wellbeing and supporting further help seeking [62]. Training educators to support children’s MH and to support appropriate parental help-seeking for children is essential given parents frequently seek help for their child’s MH problems from a preschool staff member in the first instance [27]. Information about the child from the home and education setting not only contributes to an ideal model of assessment but also may help parents recognise the need for further support for their child’s wellbeing, although further research is required.

This research has expanded the evidence base for the validity and acceptability of parent- and educator-reported PPSC and PSC-17 as utilised with young children. It presents new evidence using multi-informant reports, justifying the collection of data from educators. By validating these measures and establishing normative data, this research has increased the clinical utility of the PSC measures, enabling the early identification of young children at risk of MH problems.

## Supplementary Information

Supplementary material 1.

## Data Availability

The datasets used and analysed during the current study are available from the corresponding author on reasonable request.
