# Supplementary material for: Multi-informant reports of preschool mental health: Validation of parent and educator reports and normative data for the preschool Pediatric Symptom Checklist and PSC-17
Source: Child Adolesc Psychiatry Ment Health. 2025 Dec 9;19:138. doi: 10.1186/s13034-025-00985-3 (PMC12690794; doi:10.1186/s13034-025-00985-3)
Supplement: Supplementary file 1 — Supplementary material 1. [file 13034_2025_985_MOESM1_ESM.doc]

## Study 1

## Additional Detail about Recruitment Procedure

Participants were recruited from an Australian research panel using quotas based on census data such as household income, residential location and parental marital status. Additional quotas applied to child participants such as child gender and age to ensure a balanced sample. Caregivers could include anyone in a caregiving role such as fathers, mothers, kinship carers, or foster carers. Panel members were told a 15-minute survey was available for completion. Interested individuals completed screening questions to assess eligibility. Individuals who were eligible were provided with participant information statements, completed consent and completed questionnaires as outlined in the Method. The online questionnaires for parents of preschool-age children (3-5 years) were available for completion from December 2023 to February 2024.

At the conclusion of the questionnaires, participants received a debriefing statement and were then directed to the research panel’s website to receive compensation. Participants voluntarily participated in the anonymous study and received payment upon completion of the study.

Among the 1700 parents of preschool children who started the questionnaires, 1454 parents of preschool children completed baseline questionnaires. Digital verification was conducted by the research panel to ensure duplicate responses were not received. To maximise data integrity, data quality checks screened out any respondents who did not report data for a child in the relevant age range and/or failed attention checks.

The reported sub-sample was the number of participants who completed both the first and second assessments.

**Table 1**

*Child Ethnicity*

|  | *N* | Percent |
| --- | --- | --- |
| Caucasian (e.g., British, European) | 683 | 65.3 |
| Other or multiple ethnicities identified | 117 | 11.2 |
| Aboriginal/Torres Strait Islander | 76 | 7.3 |
| South Asian (e.g., Indian, Pakistani) | 53 | 5.1 |
| South-East Asian (e.g., Vietnamese, Filipino) | 44 | 4.2 |
| East Asian (e.g., Chinese, Japanese, Korean) | 42 | 4 |
| Polynesian (e.g., Pacific Islander, Māori) | 12 | 1.1 |
| Middle-Eastern (e.g., Egyptian, Iraqi, Lebanese) | 10 | 1 |
| African/African American | 5 | 0.5 |
| Hispanic/Latino | 3 | 0.3 |
| Total | 1,045 | 100 |

## Study 2

## Demographic Detail about Parent Participants

Parents were aged 28-47 years (*M* = 37.51; *SD* = 4.29); 95.7% identified as female and 91.5% were married or in *de facto* relationships.

The ethnicity of educators was also predominantly Caucasian (75.5%). Remaining ethnicities identified were East Asian (6.4%), South-East Asian (5.3%), South Asian (4.3%), Hispanic/Latino (3.2%), Middle Eastern (1.1%), or multiple ethnicities. No educators identified as Aboriginal or Torres Strait Islander or African/African-American.
